# Electrical Modeling of the Growth and Differentiation of Skeletal Myoblasts Cell Cultures for Tissue Engineering

**DOI:** 10.3390/s20113152

**Published:** 2020-06-02

**Authors:** Alberto Olmo, Yaiza Yuste, Juan Alfonso Serrano, Andres Maldonado-Jacobi, Pablo Pérez, Gloria Huertas, Sheila Pereira, Alberto Yufera, Fernando de la Portilla

**Affiliations:** 1 Instituto de Microelectrónica de Sevilla, IMSE, CNM (CSIC, Universidad de Sevilla), Av. Américo Vespucio, sn 41092 Sevilla, Spain; serrano@imse-cnm.csic.es (J.A.S.); jmal2d@gmail.com (A.M.-J.); pablopg@us.es (P.P.); gloria@imse-cnm.csic.es (G.H.); yufera@us.es (A.Y.); 2 Escuela Técnica Superior de Ingeniería Informática, Departamento de Tecnología Electrónica, Universidad de Sevilla, Av. Reina Mercedes, sn 41012 Sevilla, Spain; 3 Instituto de Biomedicina de Sevilla (IBIS), Campus Hospital Universitario Virgen del Rocío, Avda. Manuel Siurot, s/n 41013, Sevilla, Spain; yaiza.yuste@gmail.com (Y.Y.); spereira-ibis@us.es (S.P.); fportilla@us.es (F.d.l.P.); 4 Facultad de Física, Departamento de Electrónica y Electromagnetismo, Universidad de Sevilla, Av. Reina Mercedes, sn 41012 Sevilla, Spain

**Keywords:** oscillation-based test, skeletal muscle, stem cell differentiation, impedance spectroscopy, electrical modeling

## Abstract

In tissue engineering, of utmost importance is the control of tissue formation, in order to form tissue constructs of clinical relevance. In this work, we present the use of an impedance spectroscopy technique for the real-time measurement of the dielectric properties of skeletal myoblast cell cultures. The processes involved in the growth and differentiation of these cell cultures in skeletal muscle are studied. A circuit based on the oscillation-based test technique was used, avoiding the use of high-performance circuitry or external input signals. The effect of electrical pulse stimulation applied to cell cultures was also studied. The technique proved useful for monitoring in real-time the processes of cell growth and estimating the fill factor of muscular stem cells. Impedance spectroscopy was also useful to study the real-time monitoring of cell differentiation, obtaining different oscillation amplitude levels for differentiated and undifferentiated cell cultures. Finally, an electrical model was implemented to better understand the physical properties of the cell culture and control the tissue formation process.

## 1. Introduction

In tissue engineering, the control of tissue formation is of the utmost importance in order to form tissue constructs of clinical relevance. One of the techniques that can be used for this purpose is impedance spectroscopy, which is nowadays used for real-time monitoring of different biological processes, such as cellular proliferation, cell toxicity, and cell invasion or inflammation [1,2,3]. Impedance spectroscopy has the advantage of being a non-invasive technique (current intensity can be kept at minimum levels) and a relatively inexpensive method (only one sample or Petri plate is required for a performance curve). 

The application of impedance spectroscopy in the monitoring of the growth and differentiation of stem cells has been studied for different tissue engineering applications. Among others, the technique has been reported as a suitable tool for evaluating the functionality of human embryonic stem cell-derived retinal pigment epithelium (hESC-RPE) cells [4]. Human mesenchymal stem cell (hMSCs) development has also been analyzed with impedance spectroscopy in different works [5,6]. The impedance spectra of osteogenic treated hMSCs reported a significant increase in the magnitude of impedance compared to controls cultivated in the normal growth medium [6]. With respect to that finding, it is concluded that impedance spectroscopy is an appropriate method for non-invasive characterization of the osteogenic differentiation of hMSCs, which is relevant for quality control of cell-based implants and cell-based test systems for drug development [6].

Human adipose and human adipose-derived stem cells (hASCs) have been successfully studied and analyzed with impedance spectroscopy by Bagnaninchi et al. [7] and Nordberg et al. [8]. Impedance spectroscopy may be a useful tool to screen hASCs isolated from different donors for osteogenic differentiation potential, providing a more thorough understanding of the quality of an hASC population [8]. This technology could be incorporated into future bioreactors to track hASC through proliferation and differentiation to assist in quality control during stem cell manufacturing. To our knowledge, the impedance spectroscopy technique has never been used to monitor the growth and differentiation of skeletal myoblast stem cells, in spite of the interest of these cells in tissue engineering and the possible use of current intensity to influence and optimize the development of this cell culture. Skeletal muscle tissue engineering holds great promise for regenerative medicine. However, ex vivo cultivation methods typically result in a low differentiation efficiency of stem cells as well as the graft, which resemble the native tissues morphologically but lack contractile function. Furthermore, electrical pulse stimulation (EPS) from the central nervous system via the motor neurons is an important signal for skeletal muscle development and maturation [9]. It has been applied to induce cell clustering in cultured neural networks [10] and it is well known that physiological electrical impulses can be modeled in vitro by tuning EPS parameters such as voltage amplitude, pulse width, and frequency. However, the methodology for controlling these stimulation parameters to develop in vitro functional skeletal muscle tissues remains to be established [11]. 

In this work, impedance spectroscopy is used to study the processes of growth and differentiation of skeletal myoblasts’ cell cultures. The technique of the oscillation-based test was used, integrating the biological system into a voltage oscillator and avoiding the use of very high-performance circuitry or equipment. Initial experiments of the growth and differentiation of these specific stem cells are carried out, studying the effect of EPS in these stem cell cultures, of practical application in tissue engineering protocols. Furthermore, an electrical model is proposed to characterize the processes of growth and differentiation of stem cells, to better understand the physical properties of the cell culture and control the tissue formation process.

Our work shows the appropriateness of impedance spectroscopy to monitor tissue growth and the level of differentiation in skeletal muscle. Although originally implemented for 2D applications, the proposed system is compatible with other technologies reported in recent works, researching the most appropriate materials to implement electrodes and monitor cell and tissue evolution, for practical tissue engineering equipment [12,13,14,15]. Some works explore 2D ink-jet printed electronics, with biocompatible substrates and conductive inks as an innovative solution for implementing monitoring sensors [16]. Our oscillation-based technique could be used with the mentioned electrodes. According to [13], the main issues introduced when shifting this approach towards 3D environment are related with the distance between cells and electrodes and the electrical properties of the scaffold materials. The identification of the correct biomaterial is therefore crucial to monitor 3D cell cultures. Conductivity, biocompatibility and mechanical strength are being intensively investigated to improve the performance of engineered tissues and influence cell growth and differentiation. Other works [17] show the importance of the geometry and electronic signal applied to modify the characteristics of the formed tissue. Further work on each particular cell type is necessary in order to elucidate the optimal system to be used in each tissue engineering application.

## 2. Materials and Methods

### 2.1. Oscillation-Based Circuit

The employed circuit was based on the technique of the oscillation-based test [18,19,20], incorporating the electrode-cell impedance value (*Zcell-electrode*) as part of a voltage oscillator. The oscillation parameters (frequency of the oscillation or amplitude of the oscillation) are dependent on different biological parameters of the cell culture, such as the fill-factor (percentage of area covered by cells) or the attachment of cells to the electrode surface.

**Figure 1:**
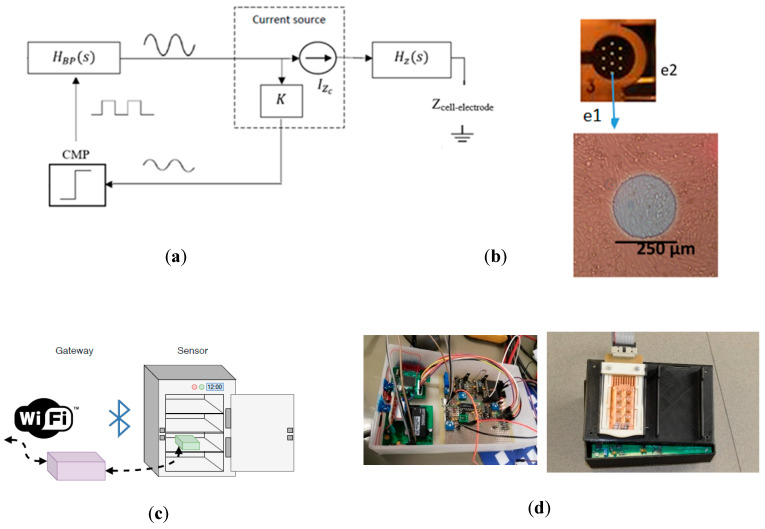
Oscillation-based circuit. (**a**) Block diagram of the circuit proposed, composed by the bioimpedance block (H_z_(s)), comparator, and bandpass filter (H_BP_(s)). The complete circuit prototype is detailed in [18,19,20]. (**b**) Detail of one of the eight wells of the 8W10E PET cultureware from Applied Biophysics [21] that were used in the experiments, where e1 is one of the 10 circular gold electrodes (the sensing area is the sum of the 10 gold electrodes) and e2 is the reference or ground electrode. Each well has an area of 0.8 cm^2^. (**c**) General diagram for the implemented prototype system. Sensor devices are located on the cell culture reactor, gathering information from the cell culture assay. This is sent to the gateway device (Intel Edison), which manages and controls the measurement acquisition system. (**d**) Photograph of the implemented experimental setup.

Figure 1 shows the schematics of the circuit used. Cell cultures were incorporated into the circuit analysis through the electrode-cell impedance value Z*cell-electrode*. This circuit works as a voltage oscillator, being characterized by its oscillation parameters—frequency of oscillation (f*osc*) and amplitude of oscillation (a*osc*) at the output voltage signal (V*out*). The circuit avoids the use of very high-performance circuitry or equipment, as well as accurate current/voltage generators, instrumentation amplifiers (IA), and exact precise demodulation circuits. The circuit was composed of a bioimpedance block, a comparator, and a band-pass filter, as shown in Figure 1. The operation of the circuit prototype with other well-established cell lines is detailed in [18,19,20].

The aim of this application was to analyze whether the circuit can be useful to characterize skeletal myoblast cell cultures’ growth and differentiation level through these parameters, with the use of an electrical model to correlate measurements and biological properties. For cell culture assays, commercial electrodes (8W10E PET) from Applied Biophysics (AB, New York, NY, USA) [21] were employed. The multi-well was composed of eight separate wells, each one containing ten circular biocompatible gold microelectrodes of 250 µm diameter in parallel and a surrounding reference electrode, which can be also seen in Figure 1. The intensity applied to the cell cultures was limited to 10 µA, although this could be changed by modifying the resistances in the bioimpedance block (shown in Figure 1).

### 2.2. Protocol of Isolation and Cultivation of Muscle Stem Cells

Rat skeletal myoblasts were obtained from Rattus Norvegicus L6 cell line (ATCC® CRL-1458™) and were cultured at 37 °C in a CO_2_ incubator at 5% at the Instituto de Biomedicina de Sevilla (IBiS, Seville, Spain). The growth medium used was Minimum Essential Medium α (12571-063, Gibco) supplemented with 10% fetal bovine serum (F7524, Sigma) and 1% penicillin-streptomycin (15140-122, Gibco). After the cells reached 85–90% confluence, they were sub-cultured using trypsin-EDTA at 0.05% (25300-062, Gibco) and 10^4^ cells were seeded in the appropriate wells of the multi-well used (wells 2, 3, 4, 6, 7, 8) with growth medium. When the specific wells reached 70% of confluence, after rinsing with phosphate-buffered saline (L0615, Linus), the medium was changed to differentiation medium and was MEMα-supplemented with 2% horse serum (S0910, Biowest) and 17.8 mM NaHCO3 (S6297, Sigma-Aldrich). Microscope images were taken with an Olympus IX-71 inverted phase microscope.

### 2.3. Experimental Growth and Differentiation of Muscle Stem Cells in the 8W10E PET Cultureware

Three sets of experiments were performed, two of them with the 8W10E PET cultureware, following the distribution of Table 1, and another experiment using traditional Petri plates, with the objective to obtain the cell culture growth control curve. All the experiments were carried out with the same protocol explained in Section 2.2.

All the cell cultures in the 8W10E PET cultureware (wells 2, 3, 4, 6, 7) were held in growth medium for control the first days. The differentiation in myotubes (wells 6, 7) was initialized by treatment with differentiation medium when 70% confluence was reached, as explained in Section 2.2., whereas the rest (wells 2, 3, 4) were held in growth medium for control. For each of these two groups of cells (cells for differentiation and cells without differentiation), one well was left without measuring impedance (wells 4 and 7) as a control in order to detect any possible effect of current intensity on stem cells. 

In wells where the impedance measurement was used (1, 2, 3, 5, 6), a limited maximum current intensity of 10 µA was applied to the cell cultures every 60 min (sample period). The cell culture growth medium and differentiation medium were also measured by the oscillating impedance system (wells 1, 5), in order to analyze any possible effect of the medium used. Table 1 summarizes the wells used in the 8W10E PET cultureware.

Temperature and humidity values were monitored during all experiments. The medium was replaced in each well every 2–3 days. During each medium change, cells were seen under the microscope, and a visual inspection and estimation of the percentage of differentiated cells were carried out. 

A western blot analysis was carried out at the end of the experiment to establish the level of expression of the anti-alpha smooth muscle actin antibody (ab5694) in the differentiated cell cultures in comparison with undifferentiated ones. Different muscle-specific proteins (Myogenin, Phos-Akt (Ser 473) and Akt) were measured in differentiated cells during different experimental days, to validate the differentiation achieved in a similar way as was carried out in [22]. 

## 3. Results

### 3.1. Study of the Effect of Current on Cell Growth and Differentiation

**Figure 2:**
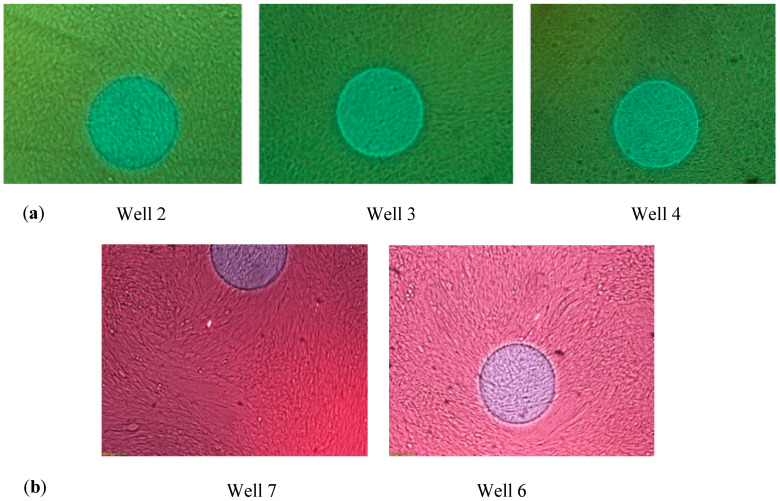
Comparison between monitored and non-monitored cell cultures. (**a**) Well 2 and Well 3 (stem cells without differentiation, using electrical impedance) and Well 4 (stem cells without differentiation, non-monitored), on day 8 of the experiment. No significant difference is observed between them. (**b**) Well 7 (stem cells for differentiation, without using electrical impedance), used as a control, on the eighth day after the start of the differentiation process and Well 6 (stem cells for differentiation, using electrical impedance) at the same time. No significant difference is observed between Well 6 and Well 7 at any moment of the differentiation process.

No significant differences were observed at the levels of current used (maximum 10 µA, applied every 60 min) between the wells where electrical impedance was used to monitor cell cultures and those used as a control without using electrical impedance (W2, W3, in comparison with W4, and W6 compared to W7, as shown in Figure 2). Good cell adhesion to the microelectrodes and to the plastic substrate was confirmed by visual inspection, in all cases.

**Figure 3:**
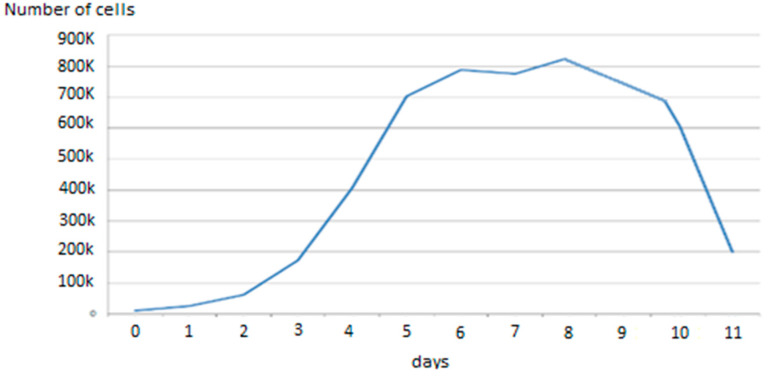
Growth curves obtained by traditional methods (cell cultures without differentiation). No significant difference between this cell culture and the cell cultures monitored on the 8W10E PET cultureware was found.

**Figure 4:**
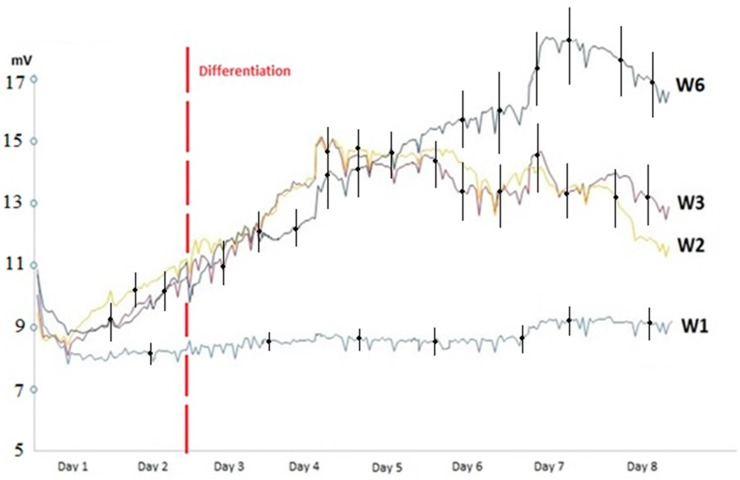
Obtained amplitude signals. Amplitude signals monitored for stem cells without differentiation (W2, W3) and with differentiation (W6). The cell culture medium was also measured (W1). It can be observed that after an initial transient regime, all signals corresponding to cell cultures started to rise, corresponding to cellular growth. After the differentiation medium was used (day 2) and after a transitory stop in the measured amplitude corresponding to a decrease in cell proliferation (with a mean delay of 0.8 days; and standard deviation of 0.1 days), stem cells following the differentiation process (W6) showed a greater increase in the monitored amplitude in comparison with the undifferentiated cell cultures (W2, W3).

This lack of difference between impedance-monitored and non-monitored cell cultures was also confirmed by comparison with the traditional cell growth cultures in traditional Petri plates using the protocol explained in Section 2.2. The number of cells, counted by visual inspection, and the cell culture behavior (see Figure 3) are similar to the cell cultures reported on the 8W10E PET cultureware (Figure 4), monitored with the oscillation-based circuit. This enables validation of the performance of cell adhesion to the presented sensing device, similar to traditional Petri plates. This validation, on the other hand, has also been well established in the different works referenced by Applied Biophysics [21] for other cell types.

### 3.2. Monitoring of the Growth and Differentiation of Myoblasts in the 8W10E PET Cultureware

Figure 4 shows the signal registered for the different cell cultures, corresponding to the amplitude of the oscillations of the circuit during the different days of the experiment. It can be seen that the behavior of muscle stem cells without differentiation (wells W2, W3) followed a similar behavior pattern to other cell cultures [5,6], compared with muscle stem cells that followed a differentiation process (W6).

The oscillation-based circuit detected initial cell growth in a similar way to other cell types [2,3]. The stem cells that did not follow a differentiation process (W2, W3) reached the confluent state in 4–5 days (as shown in Figure 4), with a good correlation between the registered amplitudes and the microscopy observations. The amplitude of the oscillations proved to be a useful parameter to determine the confluence level of the cell culture or fill factor (defined as the area occupied by cultured cells divided by the total culture area). 

**Figure 5:**
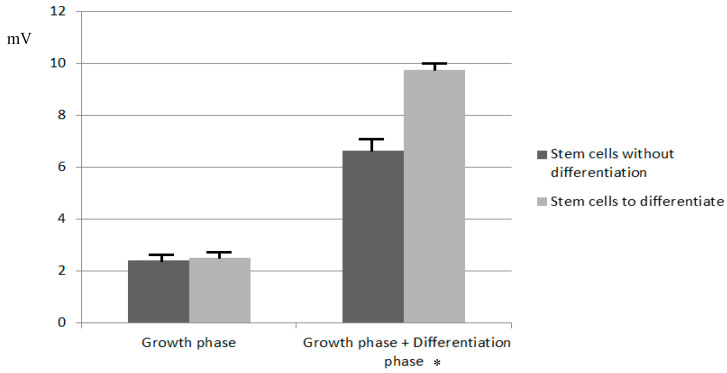
Mean measured oscillation amplitude values. Values on the left correspond with the measured difference in voltage in all cell cultures from the start of the experiment until the medium was changed to the differentiation medium. Values on the right correspond with the difference of voltage measured between the start of the experiment and the maximum amplitude measured. We can see a greater rise in amplitude measured in the cell cultures where differentiation took place. Bars indicate the standard deviation. * With a significance level set at 0.05, the *p*-value obtained was *p* = 0.031.

**Figure 6:**
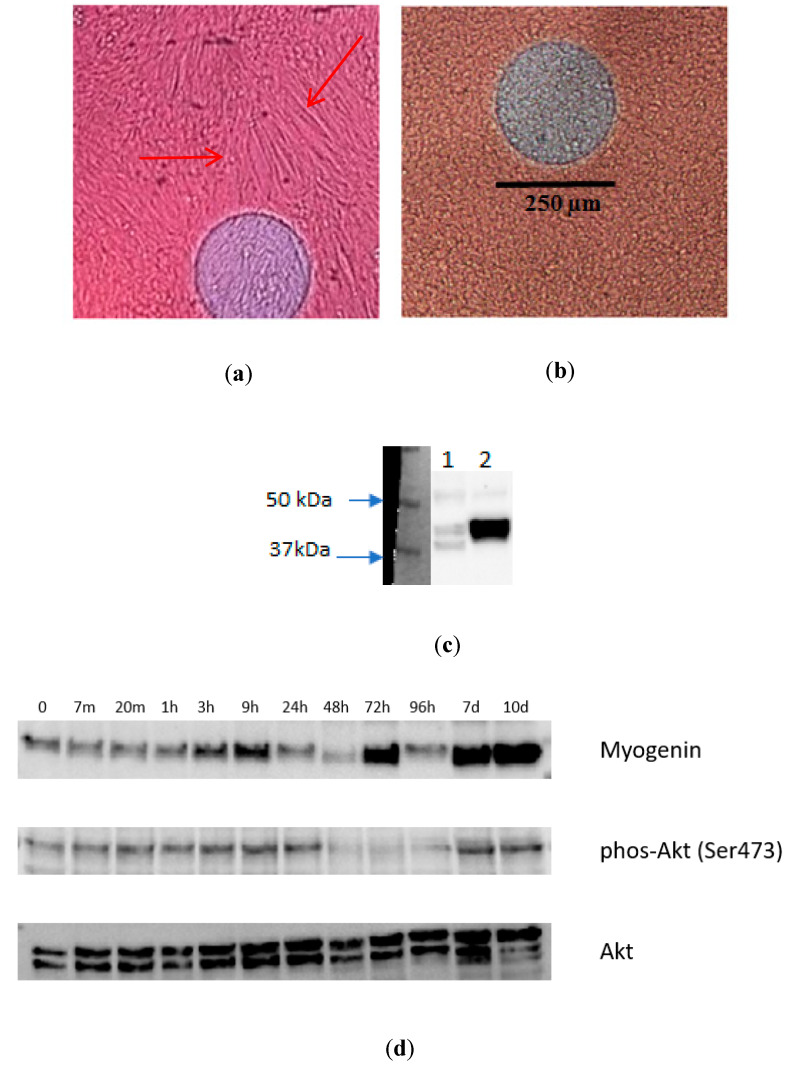
Evaluation of the cell cultures. (**a**) Microscope image of Well 6 (stem cells for differentiation) on the eighth day from the start of differentiation. Tubular structures corresponding to muscle myotubes can be observed (the arrows point at several of these structures). (**b**) Microscope image of Well 2 (stem cells without differentiation) on the eighth day from the start of differentiation in other wells. The difference between the cells that have started to differentiate and those that are not differentiating can be observed. (**c**) Level of expression of the anti-alpha smooth muscle actin antibody (ab5694, 42 kDa) in undifferentiated wells (column 1) and differentiated wells (column 2). The difference in the protein expression is in accordance with microscope images. (**d**) Level of expression of different muscle-specific proteins (Myogenin, Phos-Akt (Ser 473) and Akt) in differentiated cells, at different times during the experiment.

Stem cells cultures that changed to the differentiation medium show an initial decrease in cell proliferation after the change of medium to differentiation medium, as growth is then limited (the amplitude oscillation values stabilize for a mean of 0.8 days, between day 3 and 4, with a standard deviation of 0.1 days). However, afterward (days 4 to 7), Figure 4 shows a linear increase in the monitored amplitude for these cells corresponding to the differentiation process, which contrasts with the microscope images (where elongation and fusion among stem cells were observed), reaching a final amplitude level higher than cell cultures that did not differentiate. A more detailed analysis of the differences in the measured oscillation voltage registered is presented in Figure 5, where we show the measurement of differences of amplitude in voltage to observe the difference between differentiated and undifferentiated cultures. The microscope images can be seen in Figure 6.

Cellular growth and differentiation were observed with microscope images, as shown in Figure 6. In each medium change, cells were seen under the microscope, and photographs of all wells were taken, estimating the percentage of differentiated stem cells following traditional protocols. A good level of differentiation was observed at the end of the process (tubular structures corresponding to myotubes were observed at the end of the differentiation process; shown in Figure 6). A Western blot analysis was also carried out at the end of the experiment to establish the level of expression of the anti-alpha smooth muscle actin antibody in the differentiated cell cultures in comparison with undifferentiated ones. The results of this Western blot analysis are shown in Figure 6, demonstrating the difference between differentiated and undifferentiated cell cultures.

## 4. Discussion

### 4.1. Monitoring of the Growth and Differentiation of Myoblasts

The impedance spectroscopy technique is presented here for the first time for non-invasive and real-time monitoring of the cellular growth and differentiation processes of skeletal muscle. The technique proved to be useful for monitoring the processes of cell growth and estimating the fill factor of muscle stem cells. The oscillation-based circuit proposed successfully detected cell growth. A useful threshold could be set at a fill factor of 70%, as cells should change to the differentiation medium at this moment. The obtained growth curves correlate well with the traditional curves measured by traditional methods, as shown in Figure 3. 

Impedance spectroscopy was also useful for the real-time monitoring of cell differentiation. An initial decrease in cell proliferation was detected at the point of change of medium to differentiation medium. This decrease in cell proliferation is in accordance with other works [5]. After a few hours, a linear increase in the monitored amplitude was recorded, corresponding to the differentiation process, which contrasted with microscope images. Similar behavior is found in other works with stem cells [5,6,7]. A higher final amplitude level in differentiated cell cultures was detected. The technique could be useful for determining the degree of differentiation achieved, although more detailed tests would be needed to better characterize the differentiation process and establish the parameters of the electrical model.

No significant differences were found between cell cultures where electrical impedance was used and the control. However, higher levels of intensity could be used, which can influence the process of cellular differentiation and facilitate the development of cells, even facilitating the contraction of muscular structures, which could be of importance in the design of new bioreactors for tissue engineering.

The oscillation-based test system proved to be successful in the real-time monitoring of growth and differentiation of muscle stem cells. Our system could be used to improve the system properties with other researched electrodes, such as the 2D ink-jet printed electronics with biocompatible substrates and conductive inks presented in [16]. Although initially implemented for 2D cell cultures, the system could be adapted to 3D scaffolds, as in other works [13].

### 4.2. Electrical Modeling of the Cellular Growth and Differentiation

**Figure 7:**
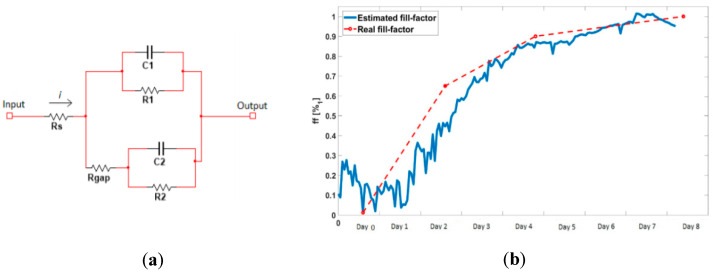
Electrical model of the cell culture and electrode system in non-differentiated wells. (**a**) The different electrical parameters (R_s_, R_gap_, R_1_, C_1_, R_2_ and C_2_) are defined following [12,16]. (**b**) Comparison of actual experimental fill factors obtained in the experiments (corresponding to the experimental visual inspection of well 2) and the fill factor obtained by the electrical model for cell culture growth (without differentiation).

An initial electrical model of the cell culture can be drafted based on some previous works [18,23] (see Figure 7a). R_gap_ models the resistance between the cell layer and the electrodes; R_s_ models the resistance between the cell layer and the reference electrode; R_1_ and C_1_ form the impedance of the area of the electrodes that are not covered by cells; and R_2_ and C_2_ model the impedance of the area of the electrodes that is covered by cells. 

Equation (1) is reproduced following the equations of the oscillation-based test model described in [18,23]:

(1) 1+HBP(s=jw)×Hz(s=jw)×HCMP(s=jw)×N(aosc)=0

For the initial and final experimental assessment of the myoblasts cell cultures, the fill factor can be defined as:

(2) $$ff=\frac{\mathrm{Acell}}{Ap}\times Ncell$$

where Acell is the average area of cells in each cell line, Ap is the well area and Ncell is the number of cells. The online estimation of the fill factor was obtained and is shown in Figure 7b. A good correlation was found between experimental measurements and the estimations of this model.

**Figure 8:**
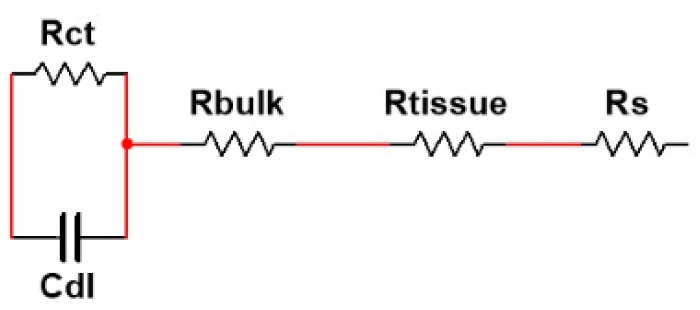
Electrical model of the cell culture and electrode system in differentiated wells. Electrical model of the differentiated tissue [18].

In cultures where differentiation takes place, a different model must be designed. When the differentiation process starts, cell growth is limited by the change of medium and all the parameters of the model remain fixed. Once the cells start to elongate and to fuse, the fill factor (percentage of electrodes covered by cells) increases, modifying some of these parameters—R_s_, R_1_ and C_2_ increase, while C_1_ and R_2_ decrease. Once the tissue is differentiated, we can adopt a similar model as the one described in [24], depicted in Figure 8, where R_gap_ is substituted by two resistances in series, R_bulk_, modeling the resistance between the microelectrode and the tissue, and R_tissue_, modeling the resistance of the tissue. R_ct_ and C_dl_ model the impedance of the area of the electrodes that are covered by the tissue [18]. If we consider *R*_bulk_ is equal to the mean R_gap_ of the cell-electrode model of the undifferentiated wells (theoretical value of *R_bulk_* is near to 1k_ohm), we can obtain the value of *R*_tissue_ from the new model when *ff* →1. The values of *R*_gap_ (for non-differentiated wells), *R*_bulk,_ and *R*_tissue_ (for differentiated wells) obtained from the two experiments are shown in Table 2. Further experiments are needed to define with more precision the electrical model and make use of this model to predict the impedance changes in the cell culture and tissue.

## 5. Conclusions

In this work, we explored the use of impedance spectroscopy for the study of the processes of growth and differentiation of skeletal myoblasts’ cell cultures. A circuit based on the oscillation-based test technique was used, converting different biological parameters of the cell culture, such as the fill-factor (percentage of area covered by cells) or the attachment of cells to the electrode surface, into the electrical oscillation parameters (frequency of the oscillation or amplitude of the oscillation), avoiding the need of an external input signal. This is translated into low-cost equipment that can be integrated with other biomedical systems for real-time analysis. 

The technique used proved to be useful for monitoring the processes of cell growth and estimating the fill factor of muscle stem cells in real-time. Furthermore, impedance spectroscopy was useful for real-time monitoring of cell differentiation, obtaining different oscillation amplitude levels for differentiated and undifferentiated cell cultures. No significant differences were found between cell cultures where electrical impedance was used and the controls, validating the obtained results. 

The proposed system is compatible with other biocompatible and conductive substrates researched to implement electrodes and monitor cell and tissue evolution in 2D and 3D scaffolds for practical tissue engineering equipment. Further work on each particular cell type is necessary in order to elucidate the optimal system to be used in each tissue engineering application. 

## 6. Patents

The work presented in this paper has been protected by a patent included on the invention registered as “Bioimpedance measurement system for wirelessly monitoring cell cultures in real-time, based on an oscillation test using integrated circuits”; register number WO2016020561A1.

## Figures and Tables

**Table 1:** Wells used in the 8W10E PET cultureware.

# Well	Culture	Impedance Measurement
1	Growth medium	Yes
2	Stem cells without differentiation	Yes
3	Stem cells without differentiation	Yes
4	Stem cells without differentiation	No
5	Differentiation medium	Yes
6	Stem cells for differentiation	Yes
7	Stem cells for differentiation	No

**Table 2:** R_gap,_ R_bulk,_ and R_tissue_ obtained in the different wells in both monitored experiments.

#Well	W2	W3	W6	W2	W3	W6
# Experiment	1	1	1	2	2	2
Differentiation	No	No	Yes	No	No	Yes
R_gap_ [Ω]	1017.6	1009.2	-	845.3	846	-
R_bulk_ [Ω]	-	-	1013.4	-	-	845.65
R_tissue_ [Ω]	-	-	158.92	-	-	114.87
